# Supplementary figures and images for: Force propagation between epithelial cells depends on active coupling and mechano-structural polarization
Source: eLife. 2023 Aug 7;12:e83588. doi: 10.7554/eLife.83588 (PMC10511242; doi:10.7554/eLife.83588)

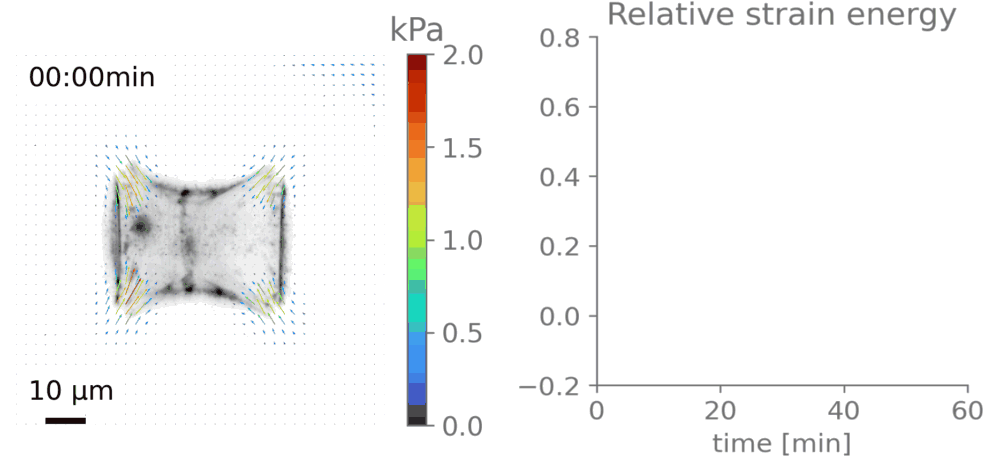

Supplement: Supplementary file 1 [file elife-83588-animation1.gif]

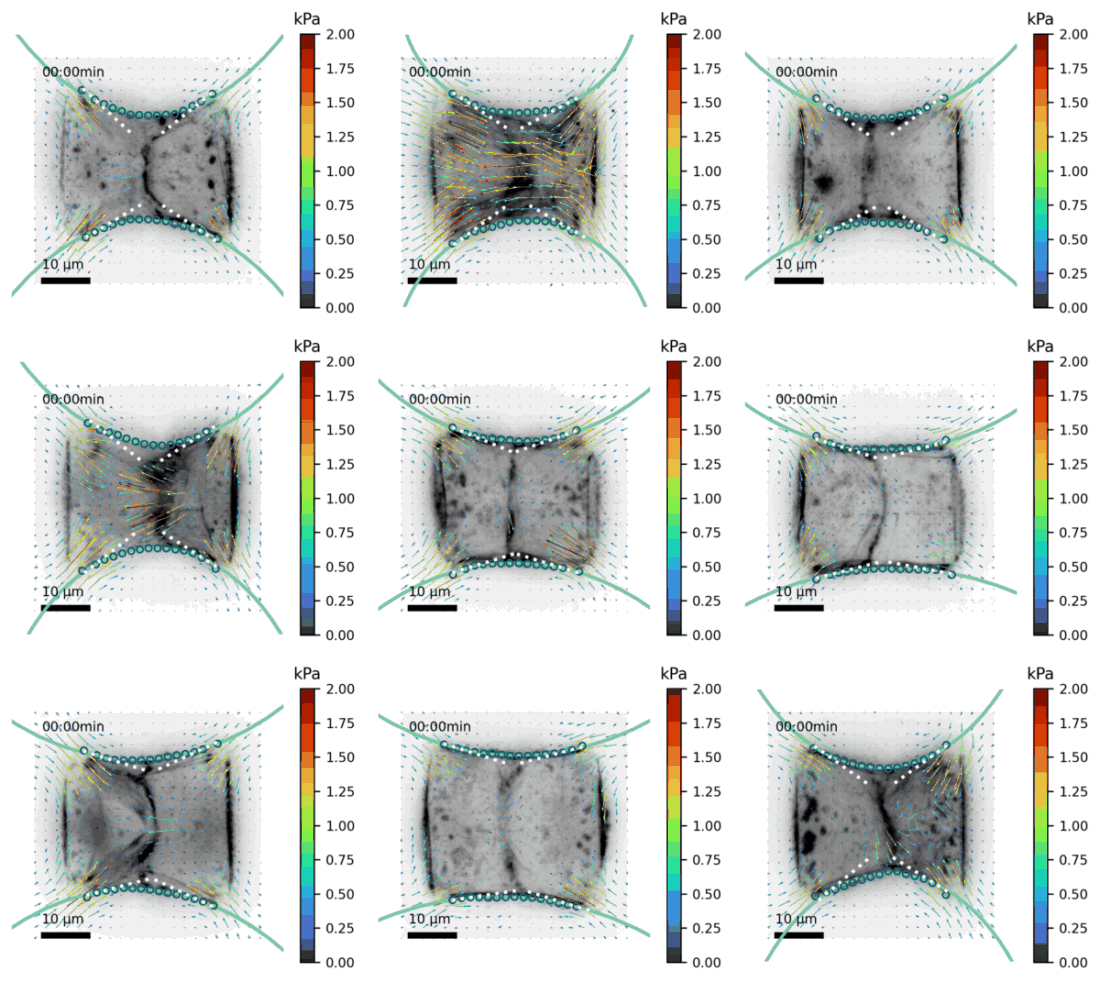

Supplement: Supplementary file 2 [file elife-83588-animation2.gif]

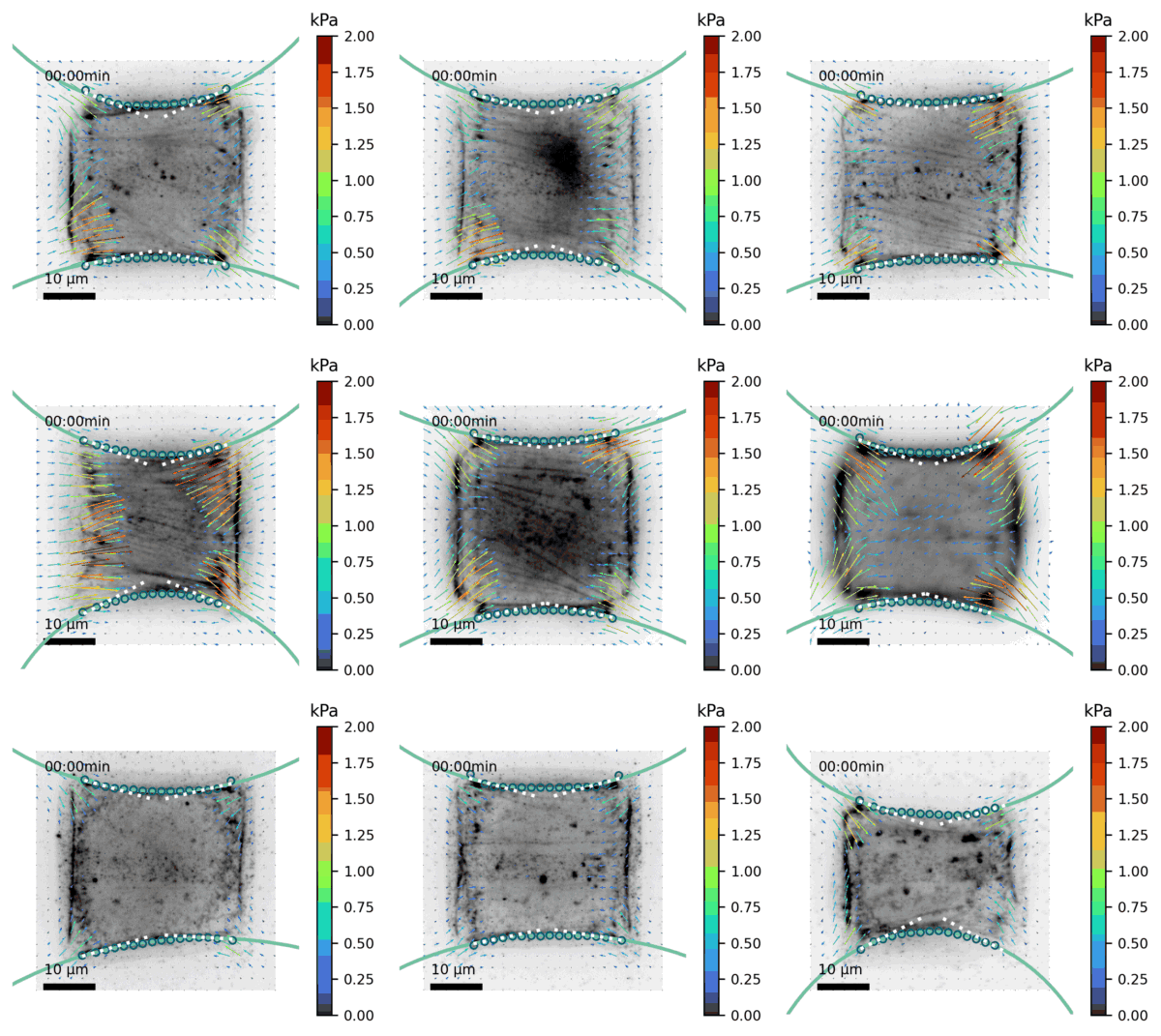

Supplement: Supplementary file 3 [file elife-83588-animation3.gif]

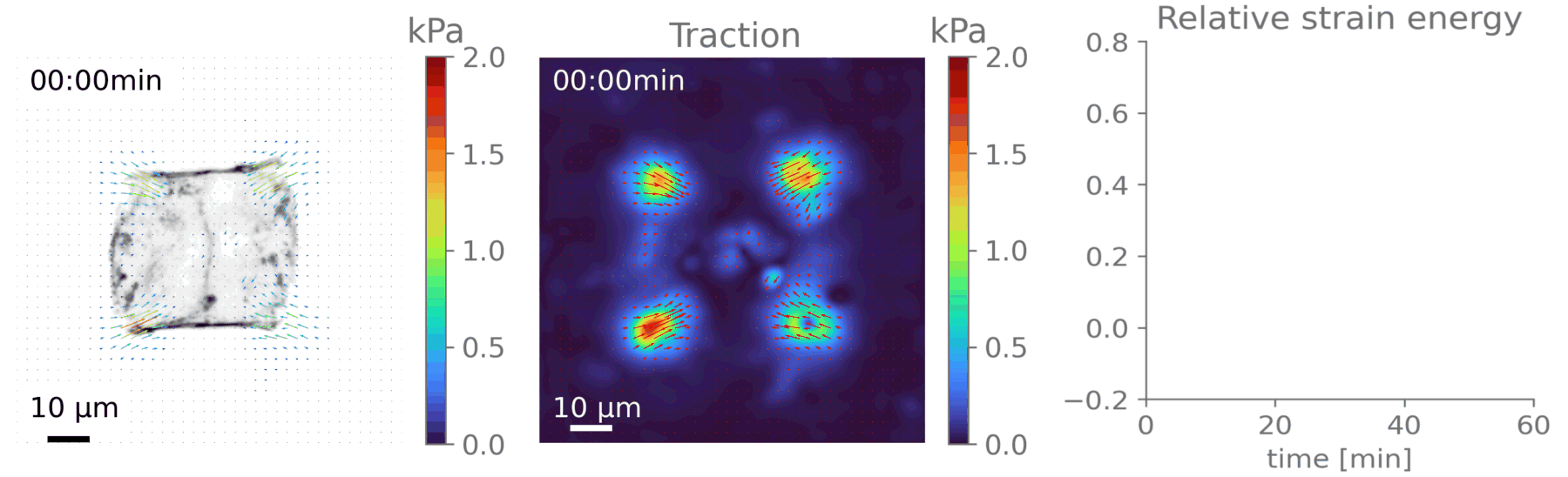

Supplement: Supplementary file 4 [file elife-83588-animation4.gif]

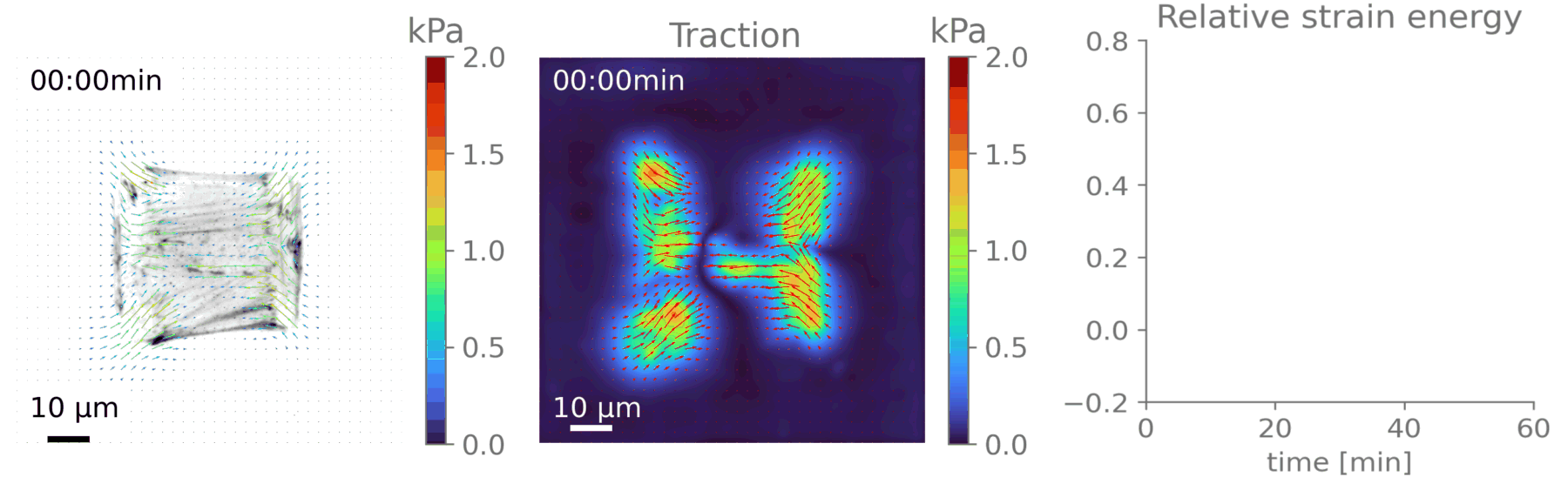

Supplement: Supplementary file 5 [file elife-83588-animation5.gif]

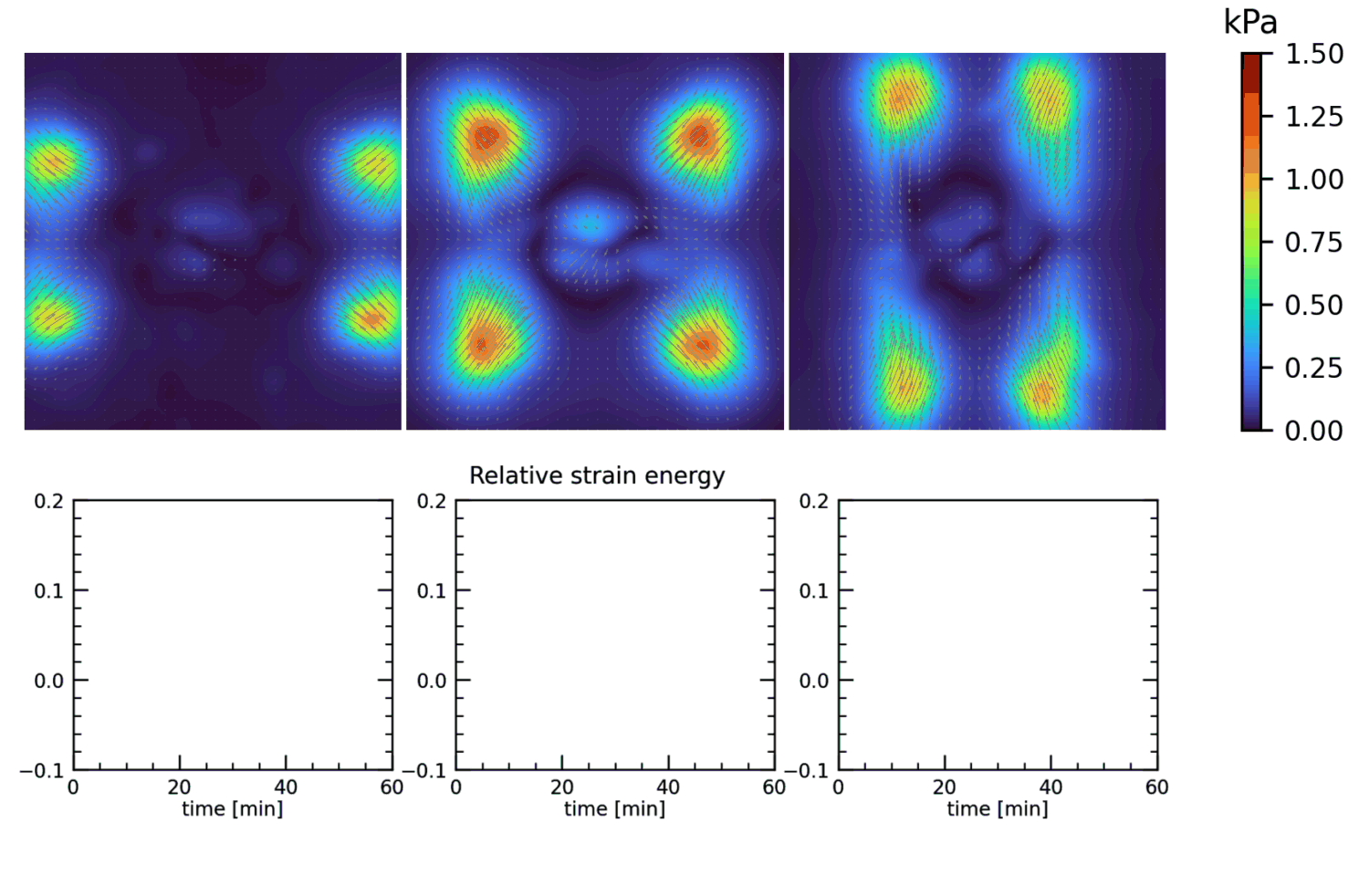

Supplement: Supplementary file 6 [file elife-83588-animation6.gif]

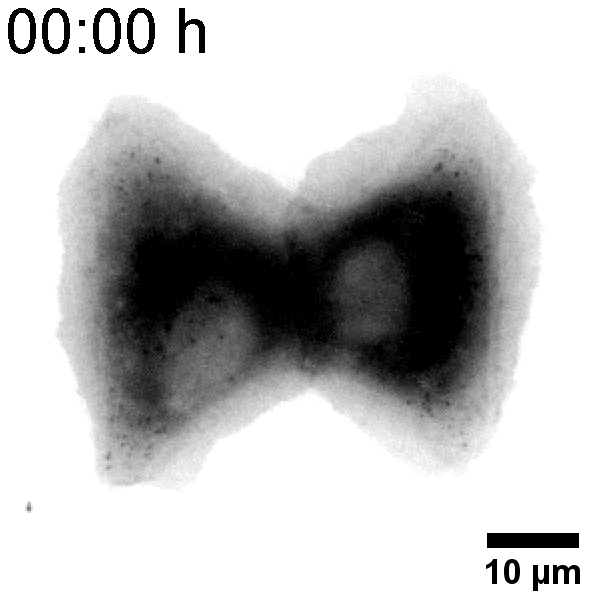

Supplement: Supplementary file 7 [file elife-83588-animation7.gif]

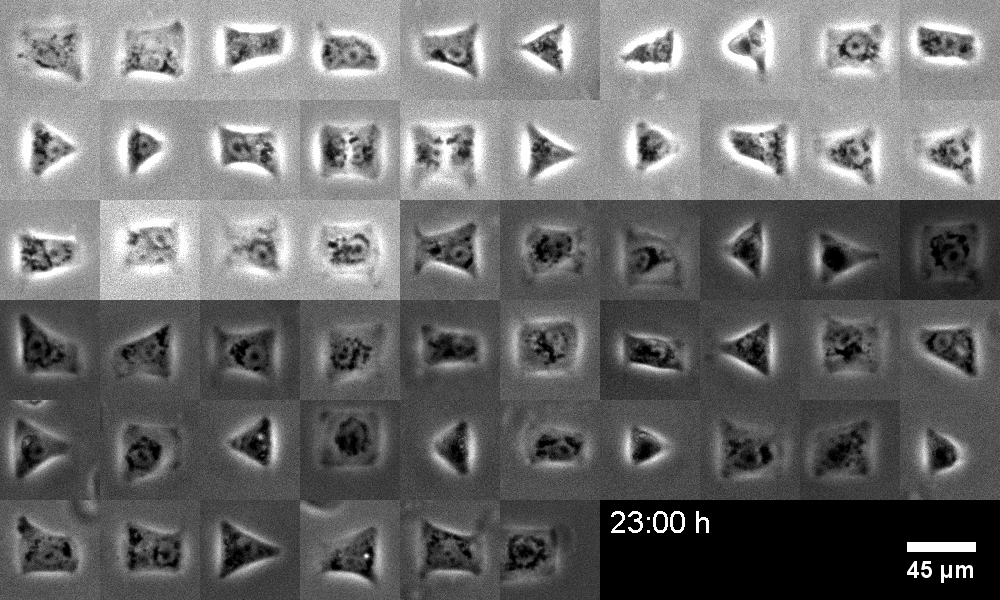

Supplement: Supplementary file 8 [file elife-83588-animation8.gif]
